# Negative dietary cation-anion difference and amount of calcium in prepartum diets: Effects on urine and serum minerals

**DOI:** 10.3168/jdsc.2023-0391

**Published:** 2023-08-19

**Authors:** K.M. Glosson, X. Zhang, K.P. Zanzalari, S.S. Bascom, A.D. Rowson, Z. Wang, J.K. Drackley

**Affiliations:** 1 Department of Animal Sciences, University of Illinois, Urbana, IL 61801; 2 Phibro Animal Health Corporation, Teaneck, NJ 07666-6712; 3 Institute of Animal Nutrition, Key Laboratory of Low Carbon Culture and Safety Production in Cattle in Sichuan, Sichuan Agricultural University, Chengdu, Sichuan, P. R. China 611130

## Abstract

Negative dietary cation-anion difference (DCAD) diets fed prepartum induce a compensated metabolic acidosis, which stimulates metabolic Ca flux before calving and decreases clinical and subclinical hypocalcemia after calving. Effects of low or high dietary Ca in these diets are unclear. Our objective was to determine the effects of inducing a prepartum metabolic acidosis and the amount of dietary Ca on urinary mineral excretion and serum mineral concentrations during the transition period in multiparous Holstein cows (n = 81). Treatment diets fed during the last 28 d before calving were (1) positive DCAD, +6 mEq/100 g of dry matter (DM), target urine pH >7.5, low dietary Ca (0.40% DM; CON); (2) negative DCAD, −24 mEq/100 g of DM, target urine pH 5.5 to 6.0, low dietary Ca (0.40% DM; ND); or (3) negative DCAD, −24 mEq/100 g of DM, target urine pH 5.5 to 6.0, high dietary Ca (2.0% DM; NDCA). Urine was sampled on −21, −14, −7, +1, +2, and +7 d relative to calving. Blood samples were collected on d −30, −21, −14, −7, −4, −2, −1, 0, 1, 2, and 4 relative to parturition. Preplanned treatment contrasts were (1) CON versus ND and NDCA, and (2) ND versus NDCA. Cows fed ND or NDCA had increased urinary volume and excretion of Ca, Cl, and SO_4_^−2^, along with decreased excretion of K. Supplementation of Ca to the acidogenic diet increased urinary excretion of Ca at d −21, but this difference lessened as cows approached parturition. Additional Ca increased prepartum urinary excretion of K and Cl. Differences in excretion decreased quickly after parturition, although urinary excretion of Cl remained greater for cows previously fed negative DCAD diets. Acidogenic diets increased serum K and Cl concentrations prepartum. Supplemental Ca decreased serum P relative to ND. During d 0 to 4, serum P and K were greater for cows fed ND or NDCA than for cows fed CON; these differences disappeared by d 4. Acidogenic diets increased serum Ca by increasing Ca flux and excretion in urine. Supplemental Ca in the acidogenic diet modestly increased Ca excretion early in the close-up period.

## Highlights

• Diets with negative DCAD increased urine Ca excretion.
• High dietary Ca further increased urinary Ca excretion prepartum.
• Blood serum potassium and chloride were increased by acidogenic diets.
• All changes reversed quickly after calving.
• Results demonstrate why acidogenic prepartum diets increase blood Ca at calving.

## Graphical Abstract

**Figure:**
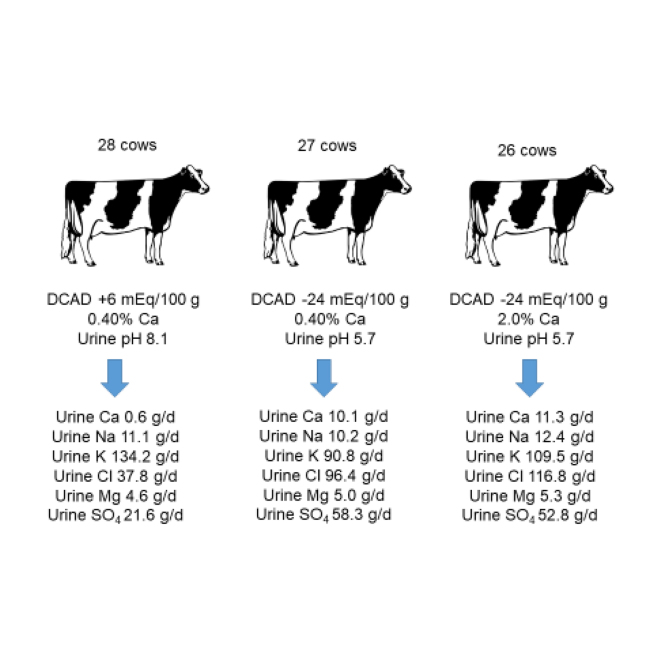
**Summary:** Pregnant Holstein cows were fed 1 of 3 diets during the last 28 days before calving. Diets were control (dietary cation-anion difference [DCAD], +6 mEq/100 g, 0.40% dietary calcium [Ca]), negative DCAD with low Ca (DCAD, -24 mEq/100 g, 0.40% dietary Ca), or negative DCAD with high Ca (DCAD, -24 mEq/100 g, 2.0% dietary Ca). Average urinary pH was 8.1, 5.7, and 5.7, respectively. Urinary excretion of Ca was increased by the negative DCAD diet and increased slightly by high dietary Ca. Acidogenic diets decreased urinary excretion of potassium and increased excretion of chloride and sulfate. High dietary Ca increased excretion of potassium and chloride. Blood serum potassium and chloride were increased by the negative DCAD diet. All changes reversed quickly after calving. Results provide information on the mode of action of acidogenic diets to maintain greater blood Ca after calving. Our data provide evidence for greater Ca in such diets.

Hypocalcemia is related to many common fresh cow metabolic disorders. Recovery to normal circulating Ca concentrations is related to reduced health events (Neves et al., 2018). Strategies to minimize hypocalcemia aim to increase metabolic flux of Ca. before parturition. Calcium flux is defined as the constant exchange of Ca within the circulating Ca pool and includes influxes from intestinal absorption, bone resorption, and renal reabsorption, and effluxes from bone calcification, renal excretion, fetal deposition, and milk production (Grünberg et al., 2011).

Maintenance dietary Ca requirements can be as low as 25 g/d, but the cow must respond quickly to the demands of lactation at the time of calving (Goff, 2014). The strategy of limiting prepartum dietary Ca through forage quality selection in a positive DCAD diet to prevent clinical hypocalcemia requires reduction of dietary Ca to below maintenance requirements, stimulating parathyroid hormone (**PTH**) release and Ca mobilization from labile bone stores. High prevalence of cation-rich forages and an insensitivity to PTH signaling in kidney PTH receptors when a positive DCAD diet is fed limit the effectiveness of this program (Liesegang et al., 2007; Goff and Koszewski, 2018).

Creating an acidogenic prepartum diet by supplementing anionic salts, primarily Cl^−^ and SO_4_^−2^, is used to prepare for the increase in Ca demand at the onset of lactation through an increase in Ca flux (Grünberg et al., 2011; Goff et al., 2014). A negative DCAD formulation causes compensated metabolic acidosis, decreasing urine pH and increasing urinary Ca excretion (Leno et al., 2017). Compensated metabolic acidosis can also directly affect Ca availability through increasing bone resorption and tissue responsiveness to hormonal signals (Liesegang et al., 2007; Rodríguez et al., 2016). In addition, increased PTH stimulates formation of calcitriol, which increases Ca absorption from the rumen and small intestines. This continuous Ca turnover creates an increased supply of available Ca that can be used at the initiation of lactation when the kidneys quickly respond by conserving Ca instead of excreting it (Grünberg et al., 2011; Megahed et al., 2018).

Effects of dietary Ca concentration in acidogenic prepartum diets remain uncertain. High dietary Ca stimulates paracellular Ca uptake from the rumen and small intestine (Goff, 2014). Amundson et al. (2018) showed that cows fed an acidogenic diet, resulting in a urine pH of 5.5 to 6.0, were the most resistant to hypocalcemia when fed the highest of 3 amounts of supplemented dietary Ca. Earlier meta-analyses indicated a quadratic relationship between risk of milk fever and dietary Ca concentration, with risk being lowest at either very low or very high dietary Ca (Oetzel, 1991; Lean et al., 2006). A more recent meta-analysis, however, concluded that the risk of milk fever increased linearly as dietary Ca increased (Santos et al., 2019).

Our hypothesis was that increasing dietary Ca in an acidogenic prepartum diet, targeting urine pH of 5.5 to 6.0, would increase Ca flux by increasing Ca loss in the urine. The objectives of our study were to evaluate the effects of 2 different prepartum dietary strategies: (1) a positive DCAD, low Ca diet compared with negative DCAD, acidogenic diets; and (2) low or high dietary Ca within a negative DCAD dietary strategy. Response variables included urinary mineral excretion and serum mineral concentrations.

All procedures involving animals were approved by the University of Illinois Institutional Animal Care and Use Committee (protocol 16115). Multiparous Holstein cows (n = 81) from the University of Illinois dairy herd were enrolled during the far-off dry period at 50 d before their expected calving date and remained on trial until 73 d postpartum. Glosson et al. (2020), Ryan et al. (2020), and Zhang et al. (2022) reported the design and partial methodology for the study, as well as productive, reproductive, and metabolic variables. Briefly, treatment diets fed as TMR during the last 28 d before expected parturition were (1) positive DCAD, +6 mEq/100 g of DM, target urine pH >7.5, low dietary Ca (0.40% DM; **CON**); (2) negative DCAD, −24 mEq/100 g of DM, target urine pH 5.5 to 6.0, low dietary Ca (0.40% DM; **ND**); or (3) negative DCAD, −24 mEq/100 g of DM, target urine pH 5.5 to 6.0, high dietary Ca (2.0% DM; **NDCA**). All cows received the same lactation diet after parturition. Diet composition and nutrient analysis were presented by Glosson et al. (2020). Cows were housed prepartum in pens with 10 sand-bedded free stalls. Calan gates (American Calan, Northwood, NH) were used to measure individual DMI. Cows calved in straw-bedded box stalls within each pen of free stalls. After calving, cows were moved to a tie-stall barn with sand-covered rubber mats.

Urine samples were collected by manual stimulation at 27, 26, 25, and 24 d before expected calving, then 3× weekly until actual calving and d 1, 2, and 7. Urine pH was determined immediately following urination using a portable pH meter and urine was then frozen at −20°C. Samples closest to d −21, −14, −7, 24 h, 48 h, and 7 d relative to actual calving were analyzed for concentrations of creatinine, Ca, Mg, Cl, K, and Na at the University of Illinois Veterinary Diagnostic Laboratory (Urbana, IL). Urinary SO_4_^−2^ was determined at the Iowa State University Veterinary Diagnostic Laboratory (Ames, IA). To estimate total daily excretion of minerals, urine output was calculated using the standard of 29 g of creatinine excreted daily per kilogram of BW in adult cows pre- and postpartum (Valadares et al., 1999).

Blood samples were obtained from the tail vein before the morning feeding every Monday, Wednesday, and Friday and daily from −7 d until calving to ensure samples were available from the actual −30, −21, −14, −7, −4, −2, and −1 d relative to calving. Postpartum samples were collected at d 0, 1, 2, and 4 after parturition. Blood was sampled into vacuum serum separator tubes (BD Vacutainer Systems), allowed to clot, and centrifuged at 1,900 × *g* for 15 min at 4°C to obtain serum. Aliquots of serum were stored at −20°C until analysis. Commercial reagents (system quantitative determination reagents, Beckman Coulter Inc.) were used in an auto-analyzer (Beckman Coulter AU680 Analyzer, Beckman Coulter Inc.) at the Clinical Pathology Laboratory in the University of Illinois College of Veterinary Medicine to measure serum concentrations of creatinine, Ca, P, Na, K, Cl, and Mg.

A total of 81 cows calved (CON = 28; ND = 27; NDCA = 26). Analytes measured in urine and blood were included in the analysis up to the time when cows were diagnosed and treated for clinical hypocalcemia (ND = 2 at 24 h; CON = 2, ND = 1 at 48 h) and data from those cows were excluded thereafter. Data from before death of 4 cows that were euthanized or died during the fresh period (CON = 2; ND = 2; NDCA = 0) were used. Thus, for postpartum data there were 27, 25, and 25 cows for CON, ND, and NDCA.

Urine and serum data were analyzed in a mixed model using SAS 9.4 (SAS Institute Inc.). Data for prepartum and postpartum periods were analyzed separately. Cow was the experimental unit. The model included fixed effects of treatment, time, and treatment by time interaction, the random effect of block, a repeated variable of sample day with cow as the subject, and covariates of initial BCS and previous milk production ranking (in quintiles). The model for prepartum serum variables included the covariate of d −30 values, when all cows were on the far-off dry cow diet. Covariates were removed from the model when *P* > 0.10. The AR(1), CS, UN, ARH(1), and CSH covariance structures were tested for repeated variables and AR(1) was selected based on the smallest values of Akaike information criterion. Model residuals were inspected for normality and homogeneity of variance. Two preplanned orthogonal contrast statements were used to compare dietary treatments. The first contrast, CON versus ND and NDCA, compared the nonacidogenic, low Ca strategy against the 2 acidogenic diet strategies (ND and NDCA). The second contrast, ND versus NDCA, compared the effect of dietary Ca concentration within a negative DCAD strategy. Significance was declared at *P* ≤ 0.05 and trends were discussed when 0.05 < *P* ≤ 0.10.

Intake of Ca did not differ between CON and ND but was greater for NDCA prepartum as planned (Glosson et al., 2020). Intakes of P, Mg, K, and Na did not differ significantly among diets either pre- or postpartum, whereas intakes of Cl and S were greater for ND and NDCA than for CON (data not shown). All cows fed ND and NDCA had urine pH values within the targeted urine pH range within 48 h after starting the treatment diet, and urine pH remained consistent throughout the prepartum period (see Figure 1 in Glosson et al., 2020). By 48 h postcalving, no cow had acidic urine (<7.0), indicating a rapid return to a normal metabolic acid-base balance when fed the lactation diet. Urinary creatinine concentration and calculated creatinine excretion (Table 1) were greater in cows fed CON (180.1 mg/dL, 22.9 g/d) compared with cows fed ND or NDCA (114.0 and 96.5 mg/dL; 22.7 and 21.4 g/d; *P* < 0.01 and *P* < 0.01). Cows fed CON excreted less urine (15.5 L/d) prepartum than cows fed ND and NDCA (20.0 and 19.3 L/d; *P* < 0.01). Cows fed ND or NDCA excreted more Ca (10.1 and 11.3 g/d) than cows fed CON (0.6 g/d; *P* < 0.01; Table 1). Calcium excretion also differed between cows fed ND and those fed NDCA (*P* = 0.05). Calcium excretion declined and the difference between ND and NDCA narrowed as cows approached parturition (treatment by time, *P* < 0.01; Figure 1).

**Table 1:** Urine creatinine and mineral concentrations and excretion by prepartum dietary treatment (Trt)

Urine	Treatment^1^			SE	Contrast,^2^*P*		*P*-value	
	CON	ND	NDCA		1	2	Time	Trt × time
Prepartum^3^								
Creatinine, mg/dL	180.1	114.0	96.5	6.8	<0.01	0.04	0.93	0.24
Ca, mEq/L	8.2	21.4	30.6	2.0	<0.01	<0.01	<0.01	0.03
Na, mEq/L	29.8	22.3	24.0	2.9	0.08	0.64	0.04	0.39
K, mEq/L	243.4	123.9	136.0	9.0	<0.01	0.30	0.02	0.98
Cl, mEq/L	71.21	120.7	158.7	8.3	<0.01	<0.01	<0.01	0.36
Mg, mEq/L	30.3	17.2	18.5	2.2	<0.01	0.62	<0.01	0.51
Sulfate, mEq/L	29.0	60.7	57.0	6.9	<0.01	0.66	<0.01	0.83
Urine volume,^4^ L/d	15.5	20.0	19.3	8.4	<0.01	0.56	0.20	0.96
Creatinine, g/d	22.9	22.7	21.4	0.25	<0.01	<0.01	<0.01	0.96
Ca, g/d	0.6	10.1	11.3	0.5	<0.01	0.05	<0.01	<0.01
Na, g/d	11.1	10.2	12.4	1.0	0.81	0.11	0.03	0.58
K, g/d	134.2	90.8	109.5	5.5	<0.01	<0.01	<0.01	0.62
Cl, g/d	37.8	96.4	116.8	4.7	<0.01	<0.01	<0.01	0.16
Mg, g/d	4.6	5.0	5.3	0.3	0.05	0.40	<0.01	0.17
Sulfate, g/d	21.6	58.3	52.8	4.5	<0.01	0.46	<0.01	0.87
Postpartum^5^								
Creatinine, mg/dL	101.4	93.8	100.6	5.4	0.57	0.54	<0.01	0.22
Ca, mEq/L	0.7	1.9	1.1	0.2	0.01	0.02	<0.01	0.03
Na, mEq/L	91.3	74.6	98.6	7.1	0.54	0.01	<0.01	0.03
K, mEq/L	175.5	164.6	195.6	10	0.70	0.03	<0.01	0.13
Cl, mEq/L	53.8	85.2	97.8	6.8	<0.01	0.12	<0.01	<0.01
Mg, mEq/L	10.8	11.5	11.0	1.2	0.72	0.76	<0.01	0.62
Sulfate, mEq/L	11.2	10.9	12.0	1.4	0.88	0.61	<0.01	0.04
Volume,^4^ L/d	25.5	29.7	24.1	16	0.23	0.12	<0.01	0.64
Creatinine, g/d	22.9	21.3	20.6	0.3	<0.01	0.05	<0.01	0.17
Ca, g/d	0.2	0.5	0.3	0.1	<0.01	<0.01	<0.01	0.13
Na, g/d	56.1	55.4	61.8	6.3	0.72	0.44	0.03	0.07
K, g/d	156.5	150.6	171.8	9.7	0.62	0.05	0.05	0.69
Cl, g/d	49.6	82.2	84.4	6.7	<0.01	0.80	<0.01	0.17
Mg, g/d	3.0	3.4	3.0	0.3	0.48	0.34	<0.01	0.29
Sulfate, g/d	12.3	12.5	12.4	1.5	0.92	0.99	<0.01	0.22

1 Diet CON = positive DCAD; ND = negative DCAD; and NDCA = negative DCAD with increased dietary Ca.

2 Preplanned contrast statements were (1) CON versus ND and NDCA and (2) ND versus NDCA.

3 Urine samples on d −21, −14, and −7.

4 Volume was calculated using 29 g of creatinine excreted per kilogram of BW.

5 Urine samples at 24 and 48 h after calving and then at d 7.

**Figure 1:**
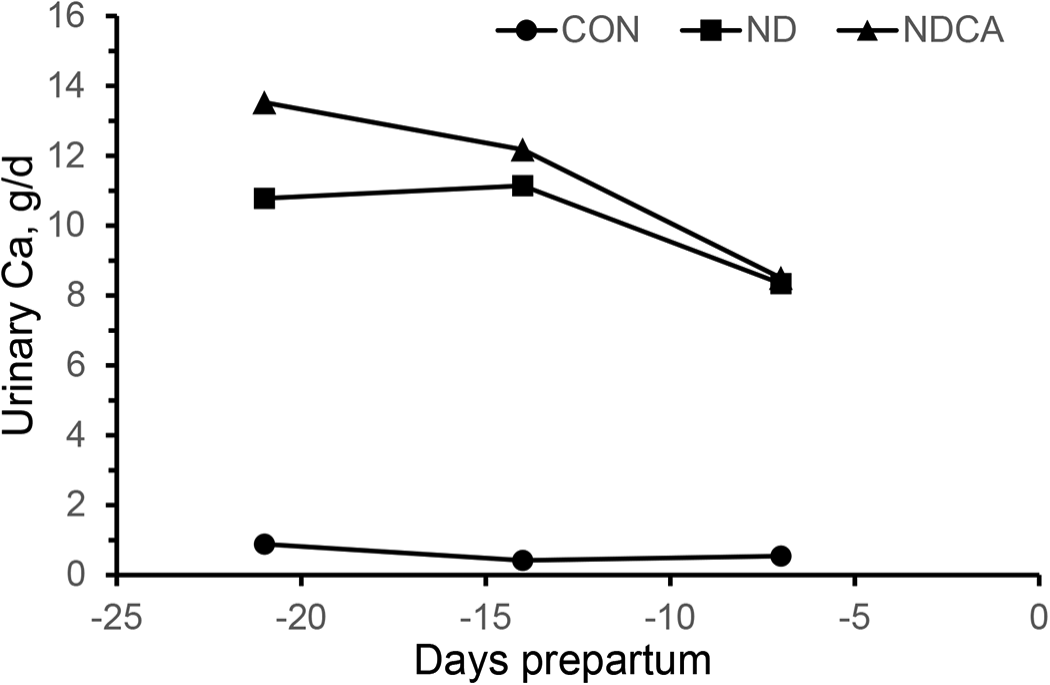
Calculated urinary Ca excretion for cows fed different prepartum diets. Diet: CON = positive DCAD; ND = negative DCAD; and NDCA = negative DCAD with increased dietary Ca. Significant effects in the model: CON versus ND and NDCA (*P* < 0.01), ND versus NDCA (*P* = 0.05), treatment by parity (*P* = 0.04), time (*P* < 0.01), treatment by time (*P* < 0.01), and initial BCS (*P* = 0.01). The largest SEM = 0.8 g/d.

Prepartum urinary Na concentration tended (*P* = 0.08) to be greater and K concentration was greater (*P* < 0.01) for CON cows than for either ND or NDCA cows (Table 1). Urinary excretion of Na did not differ. Urinary excretion of K was greater for cows fed CON than for those fed ND or NDCA, and greater for NDCA than for ND. Urinary Cl concentration and excretion were greater for cows fed ND or NDCA when compared with cows fed CON (*P* < 0.01), and were greater for NDCA than for ND (*P* < 0.01). Urinary concentration of Mg was greater (*P* < 0.01) for cows fed CON than for cows fed ND or NDCA but did not differ between ND and NDCA. Urinary Mg excretion was greater (*P* = 0.05) for cows fed ND or NDCA than for CON. Urinary sulfate concentration was greater for cows fed ND or NDCA than for CON, but did not differ between ND or NDCA. Urinary sulfate excretion was greater (*P* < 0.01) for cows fed ND or NDCA than for those fed CON, but did not differ between ND or NDCA.

Postpartum urinary Ca concentration decreased but remained greater for cows fed ND or NDCA than for cows fed CON, and greater for ND than for NDCA. Lower DMI for CON might have contributed to the difference in urinary Ca. Urinary Na and K concentrations were greater for NDCA than for ND. Urinary Cl concentration was greater for ND or NDCA than for CON. In contrast, urinary Mg and sulfate concentrations returned to normal ranges postpartum and did not differ among diets. Urinary Ca and Cl excretion decreased in all cows postpartum, but remained different between CON and ND or NDCA (*P* < 0.01 and *P* < 0.01) within the first week after calving. Urinary excretion of K differed between cows fed ND and NDCA (*P* = 0.05). Urinary excretion of Na, Mg, and sulfate did not differ among treatments.

Serum creatinine concentration was not affected by dietary treatments prepartum (Table 2). Serum Ca concentration was greater in cows fed the 2 negative DCAD diets than in those fed CON, reflecting the increase in metabolic Ca flux as a result of increased urinary Ca excretion. Serum P was lower (*P* = 0.04) in NDCA than in ND. Sodium concentration in serum did not differ among treatments, but the concentration of K increased in response to feeding acidogenic diets (*P* < 0.01). There was a treatment by time interaction for serum K concentration, where the relationship between ND and NDCA reversed before calving compared with earlier in the prepartum period. Concentration of Cl increased (*P* < 0.01) in serum of cows fed either acidogenic diet. Magnesium concentration was not affected by dietary treatments.

**Table 2:** Serum creatinine and mineral concentrations by prepartum dietary treatment (Trt)

Constituent	Treatment^1^			SE	Contrast,^2^*P*		*P*-value	
	CON	ND	NDCA		1	2	Time	Trt × time
Prepartum								
Creatinine, mg/dL	1.04	1.06	1.04	0.028	0.64	0.64	<0.01	0.72
Ca, mg/dL	9.17	9.30	9.35	0.053	0.02	0.46	<0.01	0.48
P, mg/dL	6.64	6.61	6.32	0.11	0.15	0.04	<0.01	0.56
Na, mmol/L	141.6	140.9	141.4	0.52	0.44	0.48	<0.01	0.61
K, mmol/L	4.53	4.65	4.70	0.036	<0.01	0.26	0.76	<0.01
Cl, mmol/L	103.8	106.8	106.8	0.47	<0.01	0.97	<0.01	0.28
Mg, mg/dL	2.14	2.19	2.17	0.034	0.30	0.65	<0.01	0.24
Postpartum								
Creatinine, mg/dL	1.03	1.14	1.08	0.038	0.76	0.95	<0.01	0.90
Ca, mg/dL	8.08	8.53	8.35	0.13	0.02	0.30	<0.01	0.02
P, mg/dL	5.22	5.68	5.92	0.20	<0.01	0.34	<0.01	0.22
Na, mmol/L	143.6	143.8	144.2	0.35	0.32	0.35	<0.01	0.73
K, mmol/L	4.48	4.61	4.62	0.046	0.02	0.84	0.02	0.02
Cl, mmol/L	103.4	102.8	103.9	0.44	0.99	0.08	<0.01	<0.01
Mg, mg/dL	2.03	2.11	2.22	0.047	0.37	0.38	<0.01	0.21

1 Diet CON = positive DCAD; ND = negative DCAD; and NDCA = negative DCAD with increased dietary Ca.

2 Preplanned contrast statements were (1) CON versus ND and NDCA and (2) ND versus NDCA.

After calving, serum Ca (*P* = 0.02) and P (*P* < 0.01) concentrations were greater for cows fed either acidogenic diet prepartum, with no difference due to dietary Ca (Table 2). A treatment by time interaction (*P* = 0.02) for Ca concentration showed that this difference was largely due to the Ca concentrations at calving and d 1; Ca concentrations were normalized for all treatments by d 4 (Glosson et al., 2020). Serum Na and Mg were not affected by prepartum diets, but K concentration was greater for cows fed ND and NDCA (*P* = 0.02). A treatment by time interaction (*P* = 0.02) for K concentration revealed that the difference occurred at calving and d 1, with serum K concentrations normalized for all treatments by d 2. Serum Cl tended to be greater for cows fed NDCA than for those fed ND (*P* = 0.08). A treatment by time interaction (*P* < 0.01) showed that this difference occurred on d 1 and 2.

Our data support the success of acidogenic diets to increase Ca flux by creating metabolic acidosis. Metabolic acidosis induces hypercalciuria by increasing bone resorption of Ca, reducing Ca recycling through the kidney, and increasing intestinal Ca absorption (Crenshaw et al., 2011). The increased Ca excretion in urine would represent 70% and 79% of the approximately 14.5 g in the extracellular and serum Ca pools (Goff, 2014). The amount of Ca excreted in urine would be immediately available to the cow for colostrogenesis and milk synthesis. In response, mean serum Ca concentration was greater for ND and NDCA than for CON at parturition and early postpartum (Glosson et al., 2020).

Feeding a negative DCAD diet during the dry period increases apparent absorption of dietary Ca (Schonewille et al., 1994; Vieira-Neto et al., 2021), and for cows fed NDCA, more Ca was available to be absorbed. The urinary mineral concentrations were similar to those reported in Wu et al. (2014). More Ca would be available for excretion in the urine for NDCA through the increased influx, and lack of additional dietary Ca could have limited urinary Ca excretion in cows fed ND prepartum. Chan et al. (2006) found that greater dietary Ca (1.5% of DM) nonsignificantly increased urinary Ca concentration compared with cows fed 0.99% Ca. The increase in urinary Ca excretion when more dietary Ca was provided was nearly 3 g/d on d −21, but this difference lessened as cows approached parturition. Nevertheless, the addition of dietary Ca in tandem with an acidogenic close-up diet generally was favorable or at least neutral for production, reproduction, metabolism, and the risk of adverse health events (Glosson et al., 2020; Ryan et al., 2020; Zhang et al., 2022). Our results are similar to earlier work where additional dietary Ca within an acidogenic prepartum diet, targeting urine pH of 5.5 to 6.0, improved the ability of cows to resist hypocalcemia (Amundson et al., 2018). Future meta-analyses may be more useful for determining effects of dietary Ca on production, reproduction, and health.

Excretion of Na increased slightly in response to negative DCAD, which may be due to the positive association between Na and Ca excretion (Lemann et al., 1967). Similar increases were reported by others (Grünberg et al., 2011). Excretion of Cl and S (as sulfate) increased as expected with the increased dietary supply of Cl and S as the major anions. Excretion of K was decreased by both acidogenic diets, which is a well-established response to metabolic acidosis (Constable et al., 2009). In response, serum K concentration increased for cows fed ND and NDCA diets.

Although we did not measure P loss in urine, serum P concentration prepartum was lower for NDCA than for ND. Grünberg et al. (2011) found a negative association between urine pH and serum P in cows fed a less aggressive negative DCAD diet. Serum P concentration was greater during the immediate postpartum period for cows fed ND and NDCA than for cows fed CON, similar to Ramos-Nieves et al. (2009). The increase might be related to greater bone resorption in cows fed ND or NDCA.

Feeding an acidogenic diet to target a urine pH from 5.5 to 6.0 in prepartum dairy cows primed the Ca homeostatic mechanism in late gestation through the decrease in urine pH, indicating metabolic acidosis, and an increase in urinary Ca excretion. These effects reversed quickly after parturition. Supplementing additional Ca in the prepartum diet modestly increased Ca flux and the amount of Ca excreted in the urine. Urinary excretion and serum concentrations of other minerals changed in accordance with the effects of metabolic acidosis.
